# Performance Evaluation of a Thermophilic Anaerobic Membrane Bioreactor for Palm Oil Wastewater Treatment

**DOI:** 10.3390/membranes9040055

**Published:** 2019-04-18

**Authors:** Thet Lei Yee, Thusitha Rathnayake, Chettiyappan Visvanathan

**Affiliations:** Department of Energy, Environment and Climate Change, School of Environment, Resources and Development, Asian Institute of Technology, P.O. Box 4, Khlong Luang, Pathumthani 12120, Thailand; sailaminn92@gmail.com (T.L.Y.); thusiya@gmail.com (T.R.)

**Keywords:** anaerobic membrane bioreactor, palm oil mill effluent, fouling, organic loading rate, microfiltration, thermophilic

## Abstract

Anaerobic treatment processes have achieved popularity in treating palm oil mill effluent due to its high treatability and biogas generation. The use of externally submerged membranes with anaerobic reactors promotes the retention of the biomass in the reactor. This study was conducted in thermophilic conditions with the Polytetrafluoroethylene hollow fiber (PTFE-HF) membrane which was operated at 55 °C. The reactor was operated at Organic Loading Rates (OLR) of 2, 3, 4, 6, 8, and 10 kg Chemical Oxygen Demand (COD)/m^3^·d to investigate the treatment performance and the membrane operation. The efficiency of the COD removal achieved by the system was between 93–98%. The highest methane yield achieved was 0.56 m^3^ CH_4_/kg COD_r_. The reactor mixed liquor volatile suspended solids (MLVSS) was maintained between 11.1 g/L to 20.9 g/L. A dead-end mode PTFE hollow fiber microfiltration was operated with the constant flux of 3 LMH (L/m^2^·h) in permeate recirculation mode to separate the clear final effluent and retain the biomass in the reactor. Membrane fouling was one of the limiting factors in the membrane bioreactor application. In this study, organic fouling was observed to be 93% of the total membrane fouling.

## 1. Introduction

Palm oil is a valuable oil used for food and as an ingredient for many consumable products such as cooking oil, margarine, milk fat replacer, and cocoa butter substitute. Indigenous to western and central Africa, Latin America, Malaysia, and Indonesia contribute to the greatest share (86% of global production) of the global palm oil production and export. Global consumption of palm oil increased to over 61.1 million tons in 2015 and projected to grow by 50% in 2050 (Oil World, 2016). In many cases, palm oil mill effluent (POME) comes off as a concentrated yellow waste liquid from the palm mill industry associated with sterilization, clarification, and hydro-cyclone washing processes during palm oil processing. POME has a distinct offensive odor which is characterized by high chemical oxygen demand (COD) and biochemical oxygen demand (BOD) in the range of 44,300–102,696 mg/L and 25,000–65,714 mg/L, respectively [1]. Palm oil mill effluent (POME) is normally associated with palm oil processes that typically involve the generation of large volumes of wastewater at high temperatures (80–90 °C), low pH, high BOD, COD, solids, and oil and grease as effluents [2]. Untreated or poorly treated POME released to receiving water bodies could contribute to the disruption of the aquatic ecosystem resulting in the reduction in photosynthetic activity and eutrophication. Thus, effective treatment of the POME is very crucial to meet environmental discharge standards at a reasonable economic cost. In extended applications, oil recovery during treatment may serve to reduce resource loss while also serving as a waste recovery approach in solving environmental problems.

Anaerobic, facultative, and aerobic treatments are commonly used for POME treatment. Anaerobic treatment methods have been reported to achieve over 95% BOD removal efficiency [3]. Closed anaerobic treatment options of POME have proven to be an attractive option due to the reduction of greenhouse gas to the atmosphere [4]. One of the advantages of the utilization of anaerobic processes in treating POME is the generation of biogas. Further, the performance of the system can be improved by optimizing different process conditions. Previous studies reported 93.5% COD removal from POME using combined high rate anaerobic reactors. Many studies posited that thermophilic anaerobic processes result in 25–50% higher methane yield than mesophilic conditions [5,6].

To achieve adequate solid retention in the reactor, microfiltration membranes are coupled with anaerobic systems. In this study, a Polytetrafluoroethylene hollow fiber (PTFE-HF) membrane was used coupled with an anaerobic digester in an external submerged mode. Owing to its crystalline nature and excellent thermal stability, PTFE membranes are suitable options for application in Thermophilic Anaerobic Membrane Bioreactor (TAnMBR). This study evaluated the performance of a bench scale TAnMBR by using PTFE membranes for palm oil wastewater treatment. The performance of the reactor was evaluated in terms of organic removal efficiency, biogas production rate, and methane yield. The membrane separation performance in terms of mixed liquor volatile suspended solids (MLVSS) concentration and total suspended solids (TSS) removal efficiency was evaluated. Further, the dominant membrane fouling was also investigated in this study.

## 2. Materials and Methods

### 2.1. Characterization of POME

The POME wastewater was sampled from the final discharge point of the Thai Eastern Co., Ltd., in the Chonburi district, Thailand and stored at 5 °C to prevent it from undergoing microbial degradation. POME was analyzed for the parameters of COD, BOD_5_, suspended solids, MLVSS oil and grease, total volatile solids, pH, temperature, Total Kjeldahl Nitrogen (TKN) and ammonia, following the standard methods [7].

### 2.2. Reactor Configuration and Process Flow

**Figure 1:**
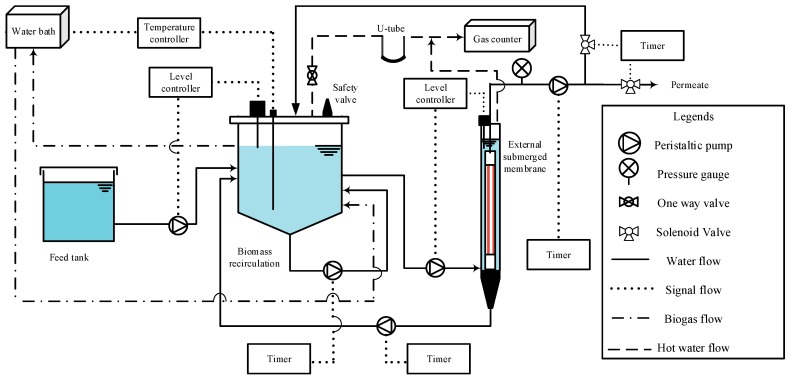
Schematic diagram of the -Thermophilic Anaerobic Membrane Bioreactor.

In this system, palm oil wastewater was used as feed water. Figure 1 presents the schematic diagram of the TAnMBR. The single stage TAnMBR was designed to operate in an automated system with a stainless steel reactor of 6 L working volume. Two biomass recirculation pumps were used for the provision of good mixing conditions. Palm oil wastewater was added to the reactor by a peristaltic pump (Master flex L/S drives, 6–600 rpm, Cole-Parmer North America, Vernon Hills, IL, USA). A level sensor immersed in the reactor controlled the level inside the reactor. The final biomass separation was carried out using microfiltration with a Polytetrafluoroethylene (PTFE) hollow fiber membrane having a pore size of 0.1 µm and an effective surface area of 0.1 m^2^. Membrane filtration was carried out with the external membrane tank. The PTFE microfiltration was operated outside-in submerged dead-end mode with negative pressure. In this study, the cleaning point was selected as −60 kPa, as per the recommendation of the membrane manufacturer. The reactor temperature was maintained at 55 °C during continuous operation.

At start-up, about 60% of the reactor volume was filled with seed sludge. During the start-up period, the inoculum was fed into the reactor with a substrate to inoculum ratio (S:I) of 1:4. The molasses was used as initial feed, and once the system became stable, palm oil wastewater was introduced gradually to the reactor. Initially, the reactor was started in the mesophilic condition of around 35 °C and increased to the thermophilic condition of 55 °C in steps of 2 °C each time. Throughout that period, pH, volatile organic acids to alkaline buffer capacity (FOS/TAC ratio), and biogas production rates were measured daily.

Following the initial start-up and stabilization of the reactor, the TAnMBR was operated in a continuous mode for 203 days including the acclimatization period of 44 days. The performance of the TAnMBR was evaluated with the same influent COD concentration of 60,000 mg/L in five stages in a continuous mode. The organic loading rates (OLR) were varied stepwise to characterize each stage. Membrane filtration was operated at a constant flux of 3 LMH (L/m^2^·h) for all the OLRs. The permeate line was divided into two paths, one for recirculation and the other for discharge. The excess permeate was returned back to the reactor. The reactor operation was automated with the solenoid valves and timer control. Membrane operations were evaluated with increments in the trans-membrane pressure (TMP) and resistance due to fouling.

The temperature was measured with a thermocouple temperature sensor. An automatic level sensor that actuates the feed pump was used to monitor and keep a constant level of feed POME in the reactor. Heat water circulation through the double wall stainless steel reactor helped to maintain the temperature.

Effluent from the anaerobic reactor was pumped into the Polytetrafluoroethylene (PTFE) hollow fiber membrane reactor for biomass separation. Two biomass recirculation pumps were used to achieve good mixing of the substrate in the anaerobic reactor. Pressure data of the system was monitored and transferred electronically with the help of pressure transducer (Trafag-Model-579225-010) and data logger (EL-USB-4). Due to membrane filtration, suspended solids and the biomass were removed and in turn, settled down in the cone-shaped bottom part of the membrane tank. The settled down biomass was recirculated back to the bioreactor by using a peristaltic pump in intermittent mode (three hours OFF and one min ON).

### 2.3. Analytical Methods

The TAnMBR performance was evaluated based on the analysis of the parameters of temperature, pH, BOD_5_, COD, TSS, NH_4_^+^-N, mixed liquor suspended solids (MLSS), mixed liquor volatile suspended solids (MLVSS), oil and grease (O&G), biogas production, and methane contentment of the biogas. The analysis of the influent and effluent parameters was carried out according to the American Public Health Association (APHA) Standard methods [7]. The temperature was measured with a thermocouple while pH was measured with a pH meter (Seven2Go^TM^ S2, Metler-Toledo AG Anlaytical, Schwerzenbach, Switzerland). The closed reflux titrimetric method was used to analyze the COD of the POME and the treated effluent (APHA, 2017-5220C) while biochemical oxygen demand was measured by the five-day BOD test (APHA, 2017-5210B). TSS was analyzed with the filter dried standard method (APHA, 2017-2540D). MLSS and MLVSS were determined by the glass fiber filter disk methods (APHA 2540D and 2540G). The titrimetric method (APHA, 2017-4500-NH3-C) was carried out for the NH_4_^+^-N analysis through a preliminary distillation step that was indicated in the 4500-NH3-B of the APHA.

O&G were analyzed with the method adapted from the 5520B Liquid-Liquid Partition-Gravimetric Method, APHA 2017. Biogas production was measured using a gas counter while methane content in biogas was analyzed using gas chromatography (Agilent 7890A, Agilent, Santa Clara, CA, USA) equipped with a thermal conductivity detector (TCD) and SUS WG-100 packed column. The FOS/TAC ratio, which measures the ratio of volatile fatty acid (VFA) to total alkalinity (TA) was measured using the titrimetric method. The PTFE-HF membrane performance was evaluated with the trans-membrane pressure variations during the operation and the resistance recovery of the membrane cleaning process.

## 3. Results and Discussion

### 3.1. TAnMBR Start-up Phase

**Figure 2:**
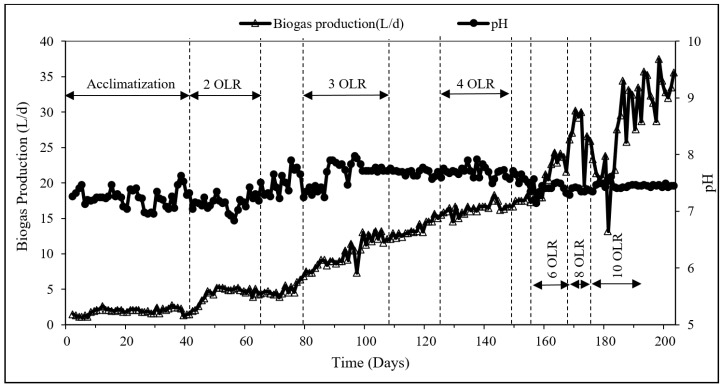
Biogas production during the operational period.

Table 1 shows the comparison of the results of the chemical and physical parameters of the POME wastewater used for the study compared to previous studies. The BOD_5_/COD ratio of about 0.7 was within the typical range of POME wastewater. The start-up phase of the TAnMBR was operated for 44 days after which the reactor was stabilized. During the start-up phase, the pH value in the reactor ranged from 6.96–7.63 with a mean value of 7.23 ± 0.16 which are within the optimum working range for anaerobic systems. During this period, biogas generation fluctuated in the range of 2.68 ± 0.54 L/d. Figure 2 illustrates the biogas production rate and the pH variation during the whole operational process including the reactor acclimatization.

To avoid a temperature shock, the temperature was increased from the mesophilic condition (35 °C) to the target temperature of 55 °C by gradual increments of 2 °C per time. It has been recommended that the operational pH in the Continuously Stirred Tank Reactor should be kept within the range of 6.5 to 8.2 since the range is considered as optimum for methanogenic microbes to produce biogas and buffering capacity against acid shock due to the overload condition [11]. The pH value in the reactor was found to be stable at 7.23 ± 0.16 and within the optimum range, during the start-up phase. Notably, about three months of acclimatization was reported in previous studies with anaerobic membrane bioreactors treating industrial wastewater [12,13]. However, this study was able to achieve steady-state conditions with a much shorter acclimatization period of 44 days.

### 3.2. Performance Evaluation of the TAnMBR during Operational Period

#### 3.2.1. Biogas Production Rate in TAnMBR

From Day 45 onwards, only palm oil wastewater was fed into the reactor starting from a loading rate of 2 kg COD/m^3^·d. With the feeding of palm oil wastewater, the biogas production increased significantly. The pH of the reactor could be maintained around 7.53 ± 0.26 over the operational period. Average biogas generation of the reactor during 2, 3, 4, 6, 8, 10 kg COD/m^3^·d organic loading rates were observed as 4.6 ± 0.5, 11.1 ± 1.8, 17.0 ± 0.7, 24.9 ± 5.2, 21.3 ± 8.1, and 32.49 ± 4.9 L/d respectively. The highest biogas production was achieved at OLR of 10 kg COD/m^3^·d, compared to the previous steps with a stable pH value of 7.5 ± 0.1. Overall, the system showed a continuous increase in the biogas production rate with increasing organic loading rates. This can be attributed to two reasons namely the increase in organic matter content and microbial activity achieved by maintaining high MLVSS in the thermophilic condition. The operating temperature is one of the main parameters that influence the biodegradation in the membrane bioreactor process [14]. POME treatment mainly followed thermophilic or mesophilic temperature conditions. The viscosity of the mixed liquor influenced by thermophilic conditions resulted in higher mass transportation and higher kinetic energy. This promoted contact between microbes and the substrate [15]. In this scenario, the thermophilic operation had a significant positive effect on higher biogas production.

#### 3.2.2. Biogas Composition

**Figure 3:**
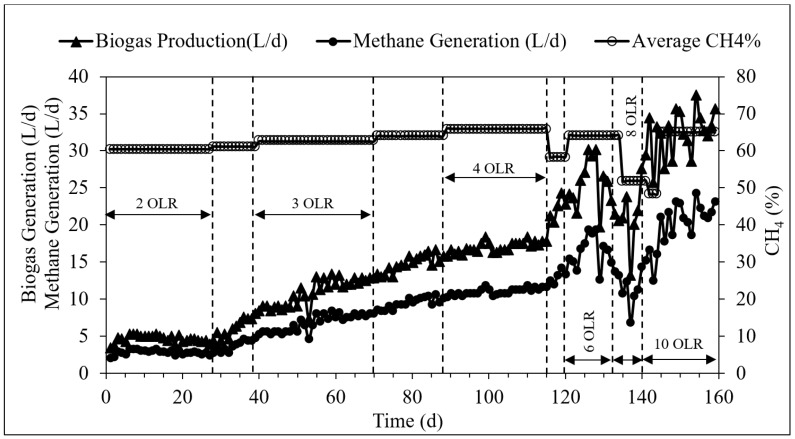
Variation of biogas production and methane generation during the reactor operation.

Figure 3 illustrates the biogas generation rate and the methane composition during the operation period. The methane generation rate measured for the OLR of 2, 3, 4, 6, 8, and 10 kg COD/m^3^·d were 2.91 ± 0.3, 6.35 ± 1.8, 11.16 ± 0.7, 15.99 ± 3.4, 11.07 ± 4.2, and 21.12 ± 3.2 L/d. A steady rise in the biogas generated by the reactor from 4.6 ± 0.5 to 32.49 ± 4.9 L/d and a similar simultaneous increase in methane production from 2.91 ± 0.3 to 21.12 ± 3.2 L/d was observed during the study.

The TAnMBR system could achieve 65.1 ± 2.2% of methane content in the biogas while treating the POME with a loading rate of 10 kg COD/m^3^·d. Methane composition in the biogas was in the range of 60.5 ± 0.5% to 65.1 ± 2.2% in almost all the testing conditions throughout the study. Compared to methane, carbon dioxide and nitrogen content of the biogas was reported less. The CO_2_ content of the biogas was in the range of 27.4 ± 2.1% to 35.6 ± 0.3% in all the OLR testing conditions. Nitrogen gas composition of the biogas showed the least compared to methane and carbon dioxide, which was reported less than 5 % for almost all OLR conditions.

#### 3.2.3. MLSS and MLVSS

**Figure 4:**
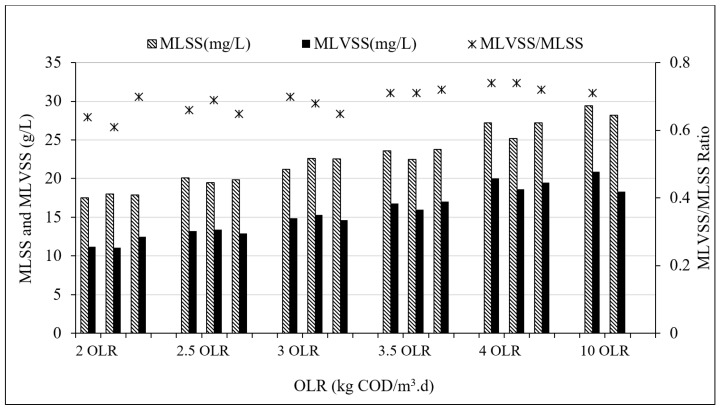
MLSS and MLVSS concentrations during the TAnMBR operation.

Figure 4 illustrates the concentration of MLSS and MLVSS during the operation period with different OLRs. As shown in this figure, MLVSS was found to increase with increased loading rate. During the first loading rate of 2 kg COD/m^3^·d, MLVSS of the reactor was observed to be about 11 g/L whereas MLSS was observed to be around 18 g/L. The MLSS and MLVSS reported a positive relationship with the organic loading rate. The highest MLVSS achieved was around 28 g/L at the highest loading rate of 10 kg COD/m^3^·d. For all the loading rates, the MLVSS/MLSS ratio was stable in the range of 0.67 ± 0.06, which indicated the stable biological activity in the reactor. Table 2 presents the comparison of MLVSS, MLSS, and MLVSS/MLSS between this study and other AnMBR studies related to POME treatment. It can be observed that the MLVSS/MLSS ratio in this study was similar to previous studies. Moreover, this operation condition and the system configuration could achieve higher MLVSS concentrations than the other POME-MBR related studies.

#### 3.2.4. COD Removal and Methanogenic Activity

The results of this study indicate methane yield was within the range of 0.19–0.56 m^3^ CH_4_/kg COD_r_ which was higher than the value reported in previous studies [17,20]. The gradual increment in methane content and biogas production indicates a higher growth rate of methanogenic microbes over acidogenic microbes. The methane yield reported in this study was higher than other AnMBR studies related to POME treatment as presented in Table 3. Moreover, it can be clearly identified that the TAnMBR reported higher overall performance than the other anaerobic POME treatment technologies.

**Figure 5:**
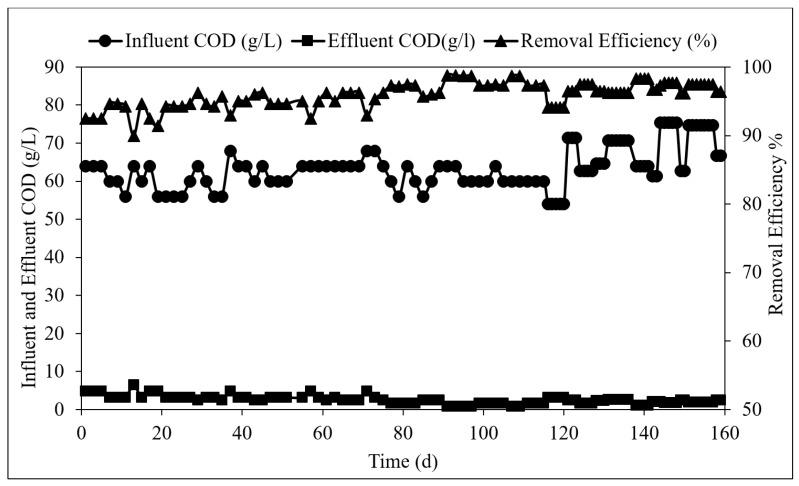
Influent and effluent COD concentrations and removal efficiency during the TAnMBR operation.

Influent and effluent COD concentrations and removal efficiency during the TAnMBR operation are shown in Figure 5. The COD removal efficiency of the system was as high as 94.3 ± 4.3% for all the OLR. Similarly, BOD removal efficiency was also observed to be in the range of 96–99% at 2–4 kg COD/m^3^·d loading rate. This indicates that the system has a high capacity to remove nearly all of the biodegradable matters present in the POME. The performance of TAnMBR under different loading rates of 2, 3, 4, 6, 8, and 10 kg COD/m^3^·d was evaluated based on the variations of the OLR against the organic removal rate (ORR).

#### 3.2.5. FOS/TAC and pH

**Figure 6:**
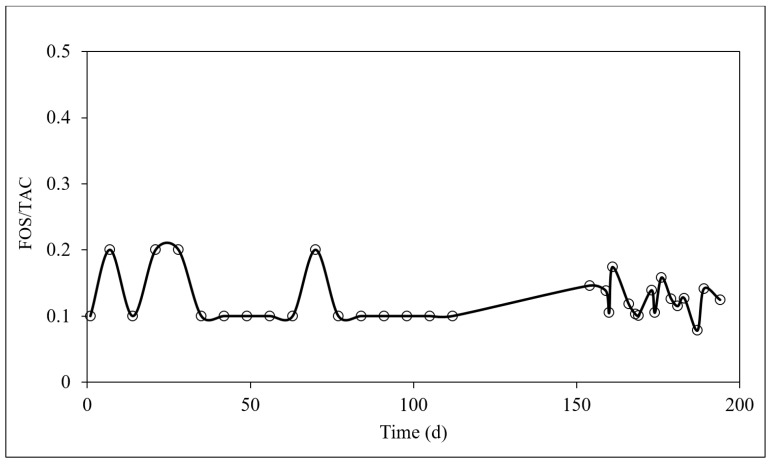
Variation of the FOS/TAC ratio during the reactor operation.

FOS/TAC was analyzed throughout the experiment for all the tested OLRs. FOS/TAC was closely monitored to observe the reactor performance. The FOS/TAC ratio was used as a diagnostic tool for the early detection of reactor failure. The ratio between VFAs and the total alkalinity concentration (FOS/TAC) coupled with pH could precisely indicate the digester performance. Figure 6 shows the FOS/TAC profile throughout the reactor operation.

In this study, the FOS/TAC ratio fluctuated between 0.1 and 0.2 over the operational period. The FOS/TAC value was reported as 0.14 ± 0.06 during the system operation. The FOS/TAC value indicated the low feeding for the microbes and the possibility to rapidly increase the feed input at that stage. Biogas production was stable between a FOS/TAC value of 0.3–0.4 based on practice. By analyzing the FOS/TAC regularly, it could provide the anaerobic system with a warning level, that alerts on the time when the system starts to experience an irreversible failure event [26]. This is because the increase in FOS/TAC ratio is closely related to the reduction of alkalinity and the accumulation of VFAs in the reactor. Figure 6 indicates that the system has the potential for stable operation for higher loading rates, beyond an OLR of 10 kg COD/m^3^·d.

### 3.3. Membrane Performance and Fouling

**Figure 7:**
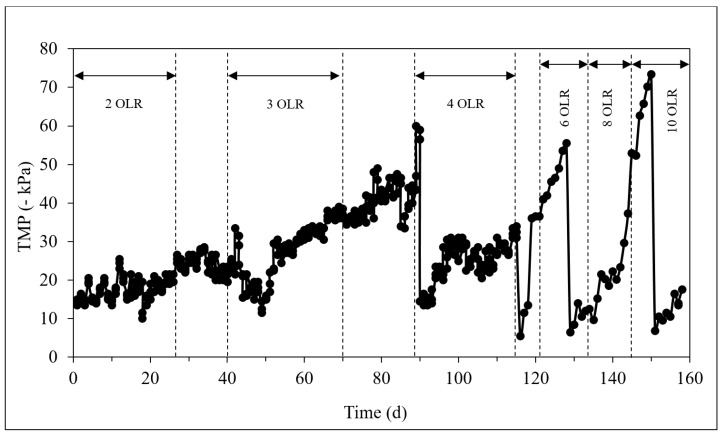
Transmembrane Pressure (TMP) variation vs. time for the TAnMBR.

The TMP variation with time was monitored to investigate membrane performance at a constant flux of 3 LMH. However, it was not possible to maintain a steady flux for all loading rates with constant TMP due to membrane fouling. The filtration resistance of the membrane was found to influence the flux. To maintain a constant flux, the flow rate was increased correspondingly by adjusting the suction rate of the pump. It was used as a parameter that indicated the requirement for membrane cleaning. The membrane cleaning procedure was conducted once the TMP increased to −60 kPa. The trans-membrane pressure over the study period was measured and illustrated in Figure 7.

As shown in the figure, the filtration cycle was able to continuously operate until day 90 without any cleaning procedure at an OLR of 2 and 3 kg COD/m^3^·d. TMP was found to gradually increase with time. On day 90, TMP increased to up to −60 kPa which is the cleaning point for the membrane. Therefore, the membrane cleaning procedure was conducted and the resistance recovery was evaluated. In this case, the major membrane fouling factor was identified as organic, which contributed to 93% of the total fouling. Moreover, the main objective of this AnMBR operation was to separate and retain the biomass in the closed system. In this scenario, the reactor could achieve nearly 100% TSS removal which helped to maintain a relatively high amount of MLVSS in the reactor. High operational stability has been reported in previous studies with AnMBRs for the treatment of different industrial wastewaters [27,28,29]. The experimental results from this study confirm the expected high operational stability, as the FOS/TAC range was reported to be less than 0.2 throughout the 203 days. Considering these benefits, it is imperative that economic analysis of TAnMBR with other relevant technologies be carried out for commercial implementation in the future.

This study on TAnMBR contributes to water sustainability through the treatment of wastewater for water reuse, along with bioenergy production. The treated water from TAnMBR is of superior quality with a reduction in germs, suspended solids, and turbidity. Notably, more than 90% removal of COD and BOD were also achieved using TAnMBR in this study. This treated water is suitable for safe discharge into the environment. Moreover, the water treated can also be reused in irrigation, industry (as cooling or cleaning water), or for domestic use (as cleaning or flushing water). This MBR technology can also be applied as a pretreatment to produce high-quality water. The ability to treat harmful pollutants present in the water makes this TAnMBR technology a promising option to ensure water availability. Adoption of this TAnMBR technology provides the opportunity to accomplish water sustainability goals by industries, in this case, the Palm Oil industry. Therefore, it is evident that this study through resource conservation will address water scarcity and ensure water sustainability.

## 4. Conclusions

This study showed that the TAnMBR coupled with PTFE-HF membrane is an effective method for treating POME. The removal efficiency of COD, BOD, and TSS was found to be over 90% in all the six organic loading rates of 2, 3, 4, 6, 8, and 10 kg COD/m^3^·d. Biogas generation rate and methane composition increased linearly with the organic loading rates. The reactor achieved over 60% of methane concentration and methane yield within the range of 0.37 ± 0.18 m^3^ CH_4_/kg COD_r_ in all the tested OLRs. The highest MLVSS of 19.6 ± 1.27 g/L was achieved at the highest loading rate of 10 kg COD/ m^3^·d. This was one of the main advantages of membrane bioreactors, which can retain the biomass within the system and increase the degradation process in order to operate at higher organic loadings. The successful application of this PTFE-HF membrane with a longer testing period in thermophilic conditions for POME treatment is the novelty of this research work. Moreover, the study reported stable performance with the organic membrane for a longer time duration of more than 200 days with less operational issues and no major problems due to membrane fouling were reported owing to the low flux adopted. Therefore, it can be concluded that TAnMBR can be an alternative way for effective POME treatment. The study suggests that the system could perform at stable conditions at higher organic loading rates.

## Figures and Tables

**Table 1:** Characteristics of the feed POME used in this study compared to previous studies.

Parameters	Unit	Current Study	Palm Oil Wastewater				
			[4]	[2]	[8]	[9]	[10]
pH	-	4.7 ± 0.1	4–5	3.4–5.2	4.75	4.5	4.5
Temperature	°C	55	-	80–90	-	55.5	-
BOD_5_	mg/L	42,670 ± 2894	25,000–65,714	25,000–65,714	30,000 ± 10,391	40,000	45,357
COD	mg/L	60,000 ± 3002	44,300–102,694	15,000–100,000	70,000 ± 7612	65,000	73,498
Oil & Grease	mg/L	7102 ± 1740	4000–9341	130–18,000	10,540 ± 1000	1500	6670.5
TS	mg/L	44,980 ± 336	40,500–72,058	11,500–79,000	-	45,000	56,279
TSS	mg/L	25,009 ± 4142	18,000–46,011	5000–54,000	28,900 ± 3065	20,000	32005.5
TVS	mg/L	37,666 ± 383	-	9000–72,000	-	26,300	41,650
Ammonia-N	mg/L	85 ± 6	35–103	4–80	-	90	69

**Table 2:** MLVSS /MLSS Ratio: Comparison between the current study and the literature.

Wastewater	Reactor Configuration	OLR (kg COD/m^3^·d)	MLSS (g/L)	MLVSS (g/L)	MLVSS/MLSS	Reference
Palm Oil Mill Effluent (POME)	AnMBR	1–11	11.76–20.8	8.9–17.68	0.76–0.85	[16]
	AnMBR	14.2–21.7	50–57	-	0.74–0.82	[17]
	Hybrid Membrane Bioreactor	10.1–11.9	15	12	0.8	[18]
	AnMBR		10.9 ± 1.2	9.2 ± 1.2	0.85 ± 0.01	[19]
	TAnMBR	2–10	17.5–29.4	11.1–20.9	0.61–0.74	This study

**Table 3:** Different anaerobic POME treatment methods and their performance.

Different Treatment Configurations	OLR (kg COD/m^3^·d)	COD Removal (%)	Methane Yield (m^3^ CH_4_/kg COD_r_)	Methane Composition (%)	Reference
Anaerobic pond	1.4	97.8	-	54.4	[21]
Anaerobic digester	2.16	80.7	-	36	[22]
Anaerobic filtration	4.5	94	-	63	[23]
Continuously Stirred Tank Reactor	3.33	80	-	62.5	[24]
Anaerobic contact process	3.44	93.3	-	63	[25]
AnMBR	1–11	96–99	0.25–0.57	-	[16]
AnMBR	14.2–21.7	91.7–94.2	0.24–0.28	-	[17]
TAnMBR	2–10	90–98.75	0.19–0.56	65.1 ± 2.2	This study
